# Remote Sensing-Based Extension of GRDC Discharge Time Series - A Monthly Product with Uncertainty Estimates

**DOI:** 10.1038/s41597-024-03078-6

**Published:** 2024-02-24

**Authors:** Omid Elmi, Mohammad J. Tourian, Peyman Saemian, Nico Sneeuw

**Affiliations:** Institute of Geodesy, University of Stuttgart, Stuttgart, Germany

**Keywords:** Hydrology, Hydrology

## Abstract

The Global Runoff Data Center (GRDC) data set has faced a decline in the number of active gauges since the 1980s, leaving only 14% of gauges active as of 2020. We develop the Remote Sensing-based Extension for the GRDC (RSEG) data set that can ingest legacy gauge discharge and remote sensing observations. We employ a stochastic nonparametric mapping algorithm to extend the monthly discharge time series for inactive GRDC stations, benefiting from satellite imagery- and altimetry-derived river width and water height observations. After a rigorous quality assessment of our estimated discharge, involving statistical validation, tests and visual inspection, results in the extension of discharge records for 3377 out of 6015 GRDC stations. The quality of discharge estimates for the rivers with a large or medium mean discharge is quite satisfactory (average KGE value > 0.5) however for river reaches with a low mean discharge the average KGE value drops to 0.33.The RSEG data set regains monitoring capability for 83% of total river discharge measured by GRDC stations, equivalent to 7895 km^3^/month.

## Background & Summary

**Fig. 1:**
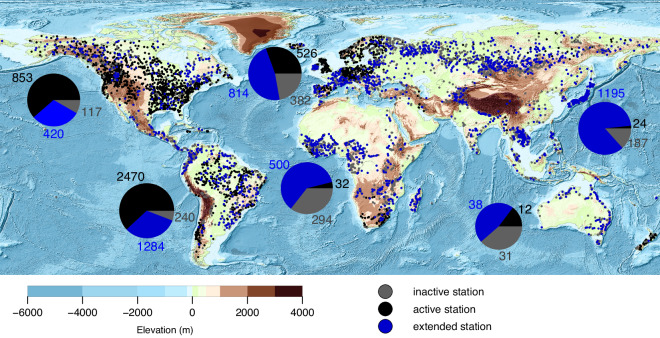
Location of the GRDC stations with a mean discharge larger than 10 m^3^/s. The GRDC stations that are active in 2015 are presented in black, the inactive stations are in grey. The stations with discharge records extended through remote sensing data are shown in blue. The pie charts’ area illustrates the river discharge on a logarithmic scale measured by active (black), inactive (grey) and extended (blue) GRDC gauges by continent. The numbers indicate the accumulated discharge over stations in km^3^/month.

**Fig. 2:**
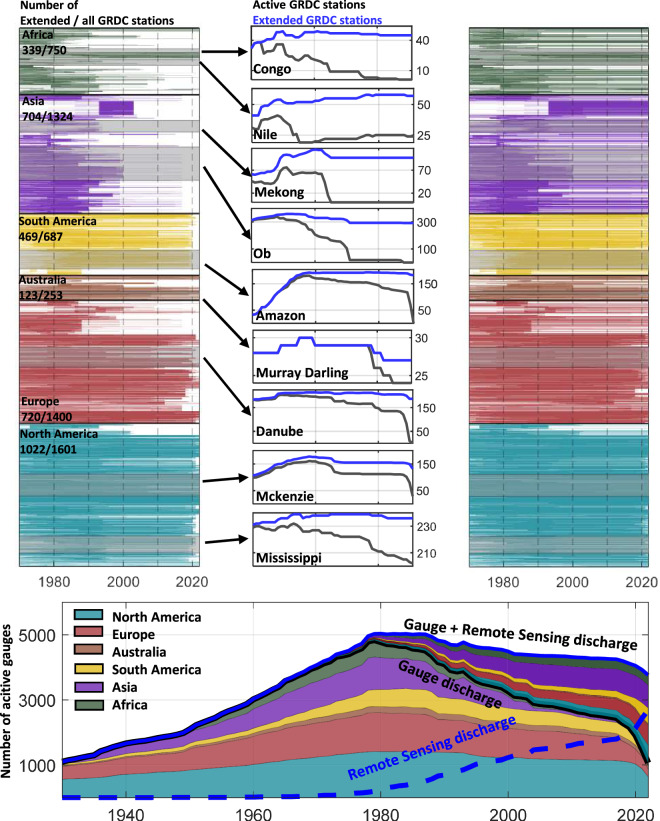
Time series in the top left panel show the activity duration of those GRDC station with a mean discharge larger than 10 m^3^/s from 1970 until 2022. The top right panel shows the length of discharge records time series in the RSEG data set. In the top middle panel, the number of active gauge stations in the GRDC and RSEG data sets in nine river basins are compared. The bottom panel shows the number of active GRDC stations by continent, together with the number of gauge stations with remote sensing-based extensions.

The global river network covers less than 1% of the Earth’s non-glacial surface and contains less than 0.01% of the Earth’s freshwater^1^, yet river water plays a multifaceted role in human well-being and natural processes as one of the most accessible sources of freshwater^2^. River discharge, which is the water volume passing a cross-section of the river at a given time, has been measured through gauge stations for more than a century^3^. Discharge measurements of global river gauges form the backbone of hydrologic science and a baseline for water resource management^4^. The Global Runoff Data Center (GRDC) collects and provides quality-controlled discharge observations worldwide through https://www.bafg.de/GRDC, that are extensively utilized by the scientific community to investigate the intricate interactions between the water cycle, climate, and ecosystems. However, over the last decades, the number of active gauges in the GRDC has decreased, resulting in a situation where most of the river basins are either poorly gauged, ungauged or have gauges that do not follow an open-data policy. Moreover, observations from publicly available gauges often come with a delay in accessibility. Consequently, out of the 6015 GRDC stations with an average monthly discharge greater than 10 m^3^/s, only 2217 stations have up-to-date measurement records after 2015. The map in Fig. 1 illustrates that a significant number of the stations providing up-to-date discharge records are concentrated in North America and Western Europe. Moreover, it is of great concern that several stations in crucial river basins in Africa and Asia have stopped providing discharge data in recent years. The number of active and up-to-date GRDC stations has continued to decrease after 2015. This becomes evident from Fig. 2, which clearly shows that only about 1000 GRDC stations have provided discharge records for the year 2022. Therefore, our understanding of the amount of water flowing through the world’s rivers is inaccurate, inhibiting a clear insight into the global spatial and temporal dynamics of river water.

Remote sensing (RS)-based discharge estimates can address the lack of a global discharge record. A promising approach is to develop empirical models, such as rating curves which establish a relationship between the ground-based river discharge and space-based measurements of a hydraulic parameter like river water level^5–9^ or river width^10–11^ or even a non-hydraulic quantity such as the ratio of surface reflectance between water and land pixels^12,13^. Once the model is developed, discharge can be estimated through remote sensing observations alone. Therefore the rating curve technique serves as a suitable method for extending or filling the gap in discharge records. With such a philosophy, numerous studies have aimed to expand the discharge estimation to a global scale. For example, Lin *et al*.^14^ estimate discharge for more than 3000 river reaches globally using the Bayesian at-many-stations hydraulic geometry (AMHG)-Manning (BAM)^15^ algorithm and the geomorphologically-enhanced variant (geoBAM)^16^. Riggs *et al*.^17^ developed a Google Earth Engine application and estimate river discharge for 28409 river reaches in North America by developing rating curves between Landsat-based river width and modeled discharge time series from Global Reach–Level A Priori Discharge Estimates for SWOT (GRADES)^18^. Riggs *et al*.^19^ developed river width-discharge rating curve models using Landsat-based river width and gauge discharge from various data centers. Using the developed model, they can extend (or fill) the river discharge time series of 2168 gauge stations (423 stations from GRDC data set). These attempts make it clear that a RS-based discharge data set exclusively developed for the GRDC stations is lacking.

**Fig. 3:**
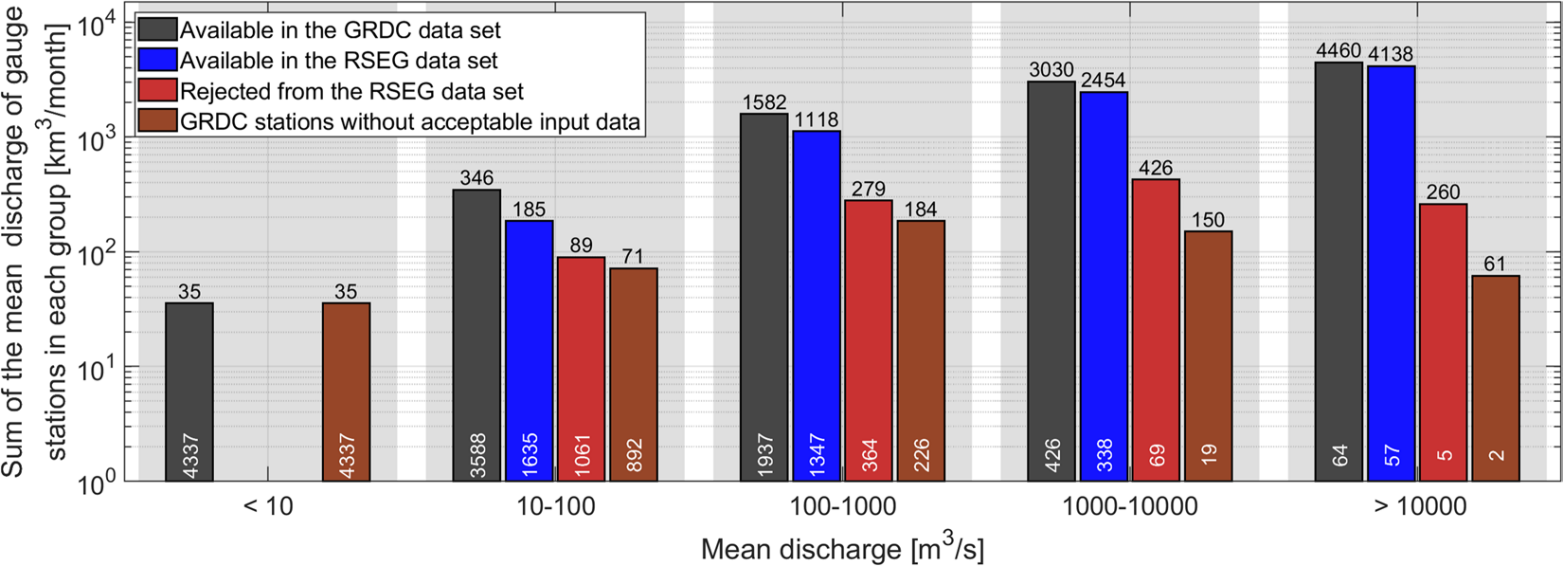
Bar graphs showing discharge (in km^3^/month) accumulated over GRDC stations in five discharge classes. Note that in the first category, those stations with mean discharge smaller than 10 m^3^/s are excluded. The blue bars depict discharge estimates available in the RSEG data sets, the red bars represent discharges that were rejected during the quality control steps, and the brown bars indicate cases without suitable remote sensing observations. The numbers on top of the bars indicate the discharge volume in km^3^/month, while the numbers on the bar feet represent the amount of stations.

In this study, we present the RS-based Extension for the GRDC (RSEG) data set^20^, delivering monthly discharge estimates accompanied by associated uncertainty. It should be noted that in the context of this paper, when we use the term “extend” we are referring to the process of utilizing Remote Sensing observations to reconstruct or estimate the river discharge at GRDC stations. This extension should not be confused with a claim of achieving an equivalence in quality or accuracy with gauge records. Relying on high-quality RS-based input data, the RSEG data set aims to overcome existing limitations identified in previous studies, thus contributing to the advancement of knowledge in this field by:

- Preserving stations with legacy discharge: by developing a discharge estimation algorithm that does not require concurrent measurements, we are able to successfully estimate discharge for stations with historical records predating the satellite era.
- Avoiding model dependence in favor of a data-driven approach: given the nonparametric nature of the employed discharge estimation algorithm, the empirical model’s form is solely determined by the observations themselves, allowing the data to directly inform and shape the model’s structure.
- Providing stochastic uncertainty of discharge estimates: the discharge estimation model incorporates stochastic uncertainty, which is quantified through the Monte Carlo estimations. This stochastic uncertainty represents the random error in the model estimates, resulting from both the remaining modeling error and the uncertainty associated with the input variables.
- Implementing several stages of supervised quality control: to ensure the accuracy of the final products, several quality control checks with and without human interactions were performed. This rigorous evaluation led to the exclusion of 1499 RS-based discharge time series from the RSEG data set, as shown in Fig. 3.
- Developing river width- or stage-discharge models and extending gauge records for 3377 GRDC stations which are significantly higher than the other studies.

In order to evaluate the performance of the data set, we validate the data against gauge discharge records over the GRDC stations where gauge- and RS-based discharge are available, which leads to the statistics listed in Table 1.

**Table 1:** Performance metrics of the RSEG data set.

Mean monthly discharge [m^3^/s]	Number of stations	KGE []	RMSE [%]	Corr. []
10–100	1635	0.29	22	0.37
100–1000	1347	0.37	22	0.45
1000–10000	338	0.49	21	0.61
>10000	57	0.62	18	0.86

The performance metrics presented in Table 1 show that the quality of discharge estimates for larger rivers (mean monthly discharge greater than 1000 m^3^/s) is quite satisfactory, with the Kling-Gupta Efficiency (KGE) value higher than 0.5 and the relative Root Mean Square Error (RMSE) below 20%. Due to the limitation of RS techniques for obtaining accurate input data for the narrow river reaches, the performance metrics significantly dropped for the river reaches with less than 1000 m^3^/s) mean monthly discharge. Despite the less-than-ideal accuracy of data for narrower river reaches, RSEG data set holds promise for a variety of research purposes:

- RSEG discharge estimates can be served as prior data for SWOT discharge algorithms. Given that most of SWOT discharge algorithms operate within the Bayesian framework, having a well-informed prior estimate, coupled with comprehensive uncertainty assessments, is of utmost significance. By integrating RSEG data and their associated uncertainties, the accuracy of SWOT discharge estimation can be improved.
- RSEG discharge estimates can be integrated into hydrological models to mitigate model uncertainty, particularly in regions where gauge data is absent or where ground-based monitoring is inadequate or no longer available.
- The RSEG data set will be an invaluable resource for climate change and environmental studies due to its ability to track discharge over an extended period. Even when data quality falls short of optimal standards in narrower rivers, RSEG data records become indispensable tools for assessing long-term trends and understanding the impacts of climate change on water resources.

## Data and Method

### Data

#### *In-situ* river discharge from GRDC data set

The GRDC has the mandate by the World Meteorological Organization (WMO) to collect, manage and distribute long-term and high-quality observations of river discharge and water level. It has done so for more than 10000 gauge stations in over 160 countries during the last 100 years. For developing the RS-based discharge estimation model, we only consider those GRDC stations with mean monthly discharge larger than 10 m^3^/s. Obtaining accurate RS-based river width or water level time series for the river reaches with mean monthly discharge less than 10 m^3^/s presents a significant challenge. These reaches are usually characterized by narrow widths or seasonal flow patterns. As a result, our analysis is limited to only 6015 GRDC stations with a mean monthly discharge larger than 10 m^3^/s. Although this limitation does reduce the number of gauge stations in the RSEG data set, the contribution of excluded stations to the global sum of mean monthly discharge is less than 0.5% (see Fig. 3). The map in Fig. 1 shows the location of the remaining GRDC stations that have been employed to develop the RS-based discharge models. Out of 6015 GRDC stations with a mean monthly discharge larger than 10 m^3^/s, only 2218 stations have updated discharge records after 2015. Note that more than 70% of these active stations are in North America or Western Europe. The GRDC discharge time series can be downloaded from https://portal.grdc.bafg.de.

#### SWORD

The SWOT a priori river database (SWORD)^21,22^ provides reach boundaries, high-resolution river centerlines, and also fixed node locations for river networks on a global scale. SWORD offers a consistent topological system for rivers wider than 30 m, along with various hydrological variables such as average surface water elevation, river reach width, and slope at the mean river flow. In this study, the river centerlines and river reach boundaries provided by SWORD are used as the base map. To simplify the terminology, we will henceforth refer to the SWORD river reach as simply *river reach*.

#### River water height time series

Over the past three decades, satellite altimetry has emerged as a valuable technique for monitoring inland water surfaces and studying the hydrological cycle^23–25^. The availability of satellite altimetry data sets has facilitated the development of numerous research initiatives and collaborations, leading to the establishment of valuable databases like Hydroweb^26^, HydroSat^9^ and Database for Hydrological Time Series of Inland Waters (DAHITI)^27^. In this study, river water height time series data were collected from the HydroSat (http://hydrosat.gis.uni-stuttgart.de) and DAHITI (https://dahiti.dgfi.tum.de) databases. To this end, virtual stations (satellite altimetry crossing the river reach) either upstream or downstream of the GRDC stations were selected in both databases, resulting in 661 stations from DAHITI and 176 stations from HydroSat, with 65 stations from both.

#### River width time series

The Global Surface Water (GSW) data set (https://global-surface-water.appspot.com) is the main source for obtaining river width time series of the river reaches corresponding to the GRDC stations. The European Commission’s Joint Research Centre (JRC) developed in the framework of the Copernicus Program a consistent monthly global surface water data set employing the entire archive of Landsat 5, 7, and 8 imagery^28^. The monthly water history product of the GSW data set constitutes the entire history of spatial dynamics of global surface water from March 1984 to December 2021 at monthly time steps and with 30 m spatial resolution^28^. Since the GSW data set is based on the Landsat mission image archive, the river extent estimates are subject to significant error mainly due to cloud contamination, sensor error, and failure in the Landsat 7 sensor after 2003.

To obtain the high-quality river reach water extent time series, the algorithm introduced by Elmi & Tourian^29^ has been applied to the GSW data set. The Elmi & Tourian algorithm aims to enhance the accuracy of river extent maps by rectifying contaminated pixels and reducing classification errors in the raw GSW data set. The algorithm tackles the problem by employing a Maximum A Posteriori estimation of a Markov Random Field model (MAP-MRF). This approach integrates both temporal and spatial constraints among pixel labels to achieve an enhanced delineation of river water extent^30,31^. The predominant Spearman correlation coefficients exceeding 0.75 between gauge discharge records and river width time series in their validation data set affirm the efficacy of the method for obtaining global-scale river width time series. To determine the effective river width for the reach, the time series of river reach water extent is divided by the length of the reach. Note that in the SWORD data set, the river reaches have a typical length between 10 and 15 km^22^. For simplicity we use the term *river width* or simply *width* instead of *effective river width* in the sequel. The river width time series used to develop RSEG data set are available through HydroSat data set (http://hydrosat.gis.uni-stuttgart.de).

In order to identify the river reach that most accurately captures the dynamics of discharge, we develop width-discharge estimation models for four distinct reaches: the reach corresponding to the GRDC station, the upstream reach, the downstream reach, as well as the average of these three reaches.

## Method

### River discharge estimation technique

Remote sensing of river discharge typically involves developing an empirical relationship between coincident measurements of gauge-based discharge and space-based river water level or width. Once established, the model enables the determination of discharge using only the space-based measurements and the model. Developing a single-stage rating curve between the ground- and space-based measurements is the most straightforward approach. However, this technique can only be used to extend the discharge records of stations where concurrent gauge- and space-based measurements are available. Also, using power-law equations to describe a river section assumes a regular geometry for the river section, which may lead to significant modeling errors. Additionally, rating curves provide imprecise estimates of discharge uncertainty due to mismodeling and coarse assumptions about input uncertainty.

**Fig. 4:**
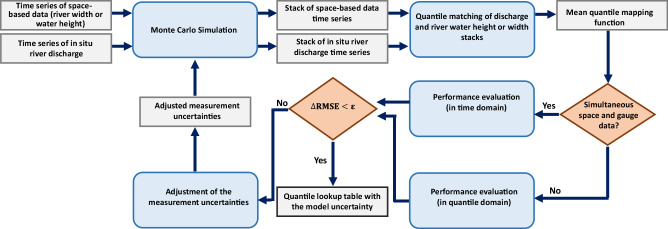
Flowchart of the stochastic quantile mapping function algorithm (adapted from Elmi *et al*.^32^).

To address the limitations of parametric rating curves, Elmi *et al*.^32^ proposed a nonparametric approach, based on Monte Carlo simulation, for developing a mapping function that transforms remote sensing observations into discharge estimates. This method sets itself apart from conventional regression techniques by not requiring simultaneous gauge-based and space-based measurements. Instead, by assuming the river bathymetry remains constant if there is any temporal gap between gauge and remote sensing observations the algorithm makes use of quantile functions of the measurements, thus eliminating the need for simultaneity in both data sets. Furthermore, this method does not assume any specific mathematical form (like linear, power-law, etc.) for the height-discharge or width-discharge model, allowing for greater flexibility and accuracy in modeling the relationship between discharge and the predictor variable in different percentiles. The flowchart in Fig. 4 describes the procedure of their algorithm. To obtain the stochastic quantile mapping function, the algorithm performs the following steps:

- Generating large numbers of river discharge and river width or height time series realizations using Monte Carlo simulation.
- Deriving a collection of quantile mapping functions by matching all possible permutations of the quantile functions of both variables.
- Estimating the mean quantile mapping function together with the uncertainty for each percentile.
- Evaluating the performance of the derived model by comparing the estimated and measured discharge of the evaluation sample performing a 3*σ* test. If available, the evaluation sample consists of simultaneous gauge- and space measurements. Otherwise, measurements from both data sets within the same quantile are included in the evaluation sample.
- Updating the measurement uncertainties with respect to the result of the 3*σ* test.
- Terminating the algorithm if the root mean square error (RMSE) from the previous step does not change significantly otherwise, the algorithm returns to the first stage.

In the initial iteration, the algorithm takes into account a multiplicative uncertainty of 10% of the signal for the input time series. As the algorithm progresses, it refines its estimates by updating the measurement uncertainties at each iteration. This iterative process continues until a termination condition is met.

Through the procedure, the algorithm generates a stack of quantile mapping functions by propagating the input measurements based on their respective uncertainties. The distortion observed in the collection of mapping functions illustrates the model’s accuracy in estimating discharge at various percentiles. The discharge estimation model uncertainties are later exploited to obtain the uncertainty of the RS-based discharge estimates. Once the model is developed, the discharge value, along with its associated uncertainty, can be estimated using solely the predictor variable.

### Algorithm implementation

The procedure of developing river discharge estimation models begins with assessing the feasibility of employing remote sensing data to extend the GRDC records. The discharge estimation technique is not constrained to stations with simultaneous gauge- and space-based data. However, it requires an adequate number of measurements in both data sets to sufficiently represent the statistical distribution of the variable. Therefore, those GRDC stations with less than three years of discharge records or insufficient observations for the predictor variable (water height or river width) after 1970 are excluded from the analysis. Due to the constraints posed by the inter-track distance of satellite altimetry missions, it is unfeasible to obtain water level time series for all the GRDC stations. As a result, in this study, we only have altimetric time series for 766 out of 6015 GRDC stations. On the other hand, satellite imagery missions have global coverage, so theoretically, river width time series for any reach can be contained using satellite images. However, due to cloud contamination and also the complex shape of the reaches, a total of 4743 GRDC stations were identified where width measurements were available for a minimum of three years. Among these, 639 GRDC stations have both river width and water height time series available. After preparing the input data sets, the river discharge algorithm is applied to develop the quantile mapping function for each GRDC station. Depending on the available remote sensing time series, the discharge estimation model can be developed using river width or water level time series, or both.

**Fig. 5:**
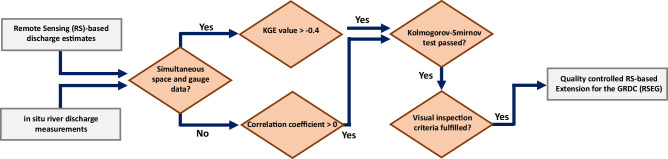
Flowchart of the quality assessment procedure for the remote sensing-based discharge estimates. KGE values are calculated for simultaneous time series of GRDC and remote sensing-based discharge, while the correlation coefficient is calculated for mean monthly values of nonsimultaneous time series of gauge and remote sensing-based discharge.

After developing the models, the discharge records of each GRDC station are further extended by incorporating remote sensing data. Subsequently, a quality assessment procedure (Fig. 5) is implemented to ensure the reliability of the estimated discharge records. This step involves performing statistical tests and also visual inspection to identify and exclude discharge estimates with low quality or potential anomalies. The quality assessment starts with calculating KGE^33^ between the observed and estimated discharge values. KGE is a comprehensive statistical metric to assess the performance of hydrological models by considering correlation, bias, and variability in the simulated data compared to observed data. If we consider the mean flow as a benchmark predictor, then any model with a KGE value between −0.4 and 1 has an acceptable performance. The closer the KGE value is to 1, the better the model’s performance. A KGE value of 1 indicates a perfect agreement between estimated and observed discharge^34^. In order to ensure the quality of the final results, estimated discharges with a KGE value lower than −0.4 are removed. This assessment focuses solely on GRDC stations where both gauge-based and RS-based observations are available simultaneously. For GRDC stations that lack simultaneous gauge and space-based observations, we compare the behavior of the monthly mean of gauge and space-based discharge (obtained from two different time periods) by calculating the correlation coefficient values. Discharge estimates with a negative correlation coefficient are excluded to retain only high-quality RS-based discharge estimates. In the next step, to ensure the compatibility of the distribution of both estimated and measured discharge values, we perform a Kolmogorov-Smirnov (KS) test. The null hypothesis of this test is whether both estimated and measured discharge represent the same statistical distribution with a 5% significant level. Finally, we perform a visual inspection to identify any possible anomalies, and issues that might not be apparent through quantitative methods. In the visual inspection procedure, we consider several key aspects to keep the estimated discharge in the data set; unusual long-term patterns (e.g. trend or cyclical behavior), any notable variations or anomalies (sudden spikes, drops, or erratic fluctuations), the existence of outliers or unusual extreme values. In case of possible non-stationarity of estimated and measured discharge, the station would probably fail the KS test. However, If non-stationarity only appears in the form of a trend, it may pass the KS test but would be detected and removed during the visual inspection.

In the case of width-based discharge, usually, multiple solutions are available since discharge estimation models are developed for upstream and downstream reaches, as well as the average of three reaches. During the quality control steps, only one set of discharge estimates with the best performance remains in the data set. In the case of the availability of height-based discharge estimates obtained from the DAHITI and HydroSat altimetric time series, we choose the one with a better performance in the quality assessment procedure.

Through the quality assessment procedure, discharge estimates based on remote sensing approaches for 1499 GRDC stations were rejected and subsequently removed from the data set. As illustrated in Fig. 3, over 95% of the rejected discharge values belong to GRDC stations with a mean discharge of less than 1000 m^3^/s. This highlights the inherent challenge in accurately estimating discharge using space-based methods, particularly for narrower rivers. In contrast, the performance of RS-based approaches in estimating discharge for GRDC stations with a mean discharge exceeding 1000 m^3^/s is remarkably reliable. Out of the 426 GRDC stations, with an average monthly discharge ranging from 1000 to 10000 m^3^/s, only 19 stations lacked sufficient satellite observations, and the RS-based discharge estimates for 69 stations are rejected through the quality assessment procedure. Consequently, for 338 GRDC stations, the RS-based discharge estimates are accepted and included in the RSEG data set. The significance of RS-based estimates becomes even more pronounced for GRDC stations with an average monthly discharge greater than 10000 m^3^/s, with 57 out of 64 stations having available RS-based discharge estimates. Notably, approximately 80% of the global river flow, equivalent to 7490 km^3^/month, is represented by the GRDC stations with a mean discharge of each exceeding 1000 m^3^/s. About 88% of this water volume (equivalent to 6592 km^3^/month) is quantified through RS-based discharge estimates within our RSEG data set.

**Fig. 6:**
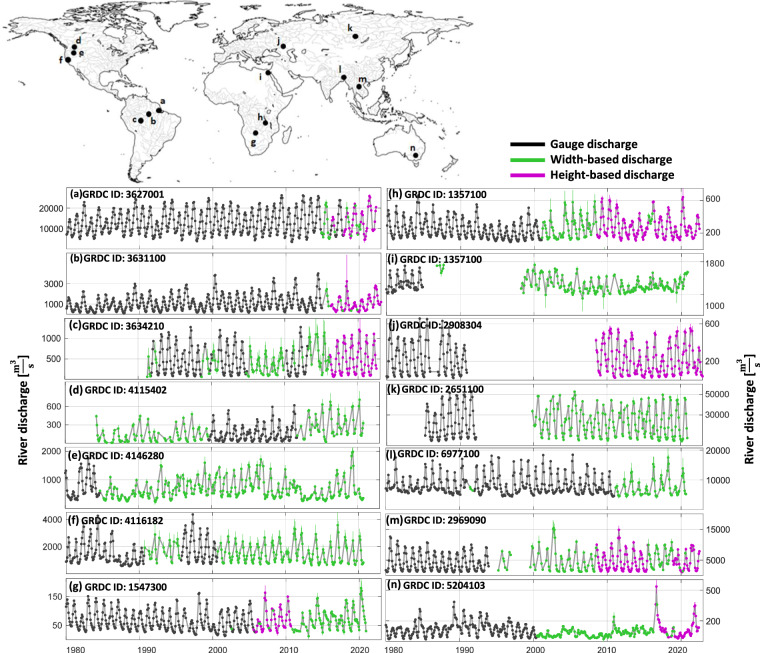
Examples from the RSEG data set showing width-based and/or height-based discharge time series. All values are provided with an error bar.

Figure 6 provides several examples of GRDC discharge time series that have been extended through remote sensing observations. In these examples, the gauge stations have stopped delivering discharge records. However, we extend the discharge time series by merging the RS-based discharge estimates. Since satellite images provide global coverage, river width-based discharge estimates are available for most of these examples. As mentioned, for 639 GRDC stations, both height-based and width-based discharge estimates are available. When both estimates are concurrently available at a given time, we prioritize the height-based discharge in the final solution, since the discharge estimates obtained through water height measurements are generally more accurate than those derived from river width.

Time series like Fig. 6(b,g,m,h) are some examples of stations where both river width- and height-based discharge estimates are available. In addition to extending the gauge discharge records, RS-based discharge estimates can fill gaps in gauge records, as shown in the time series in Fig. 6(a,c,f). Furthermore, RSEG data set provides discharge estimates for periods prior to the establishment of gauges, as demonstrated in Fig. 6(d,c).

**Fig. 7:**
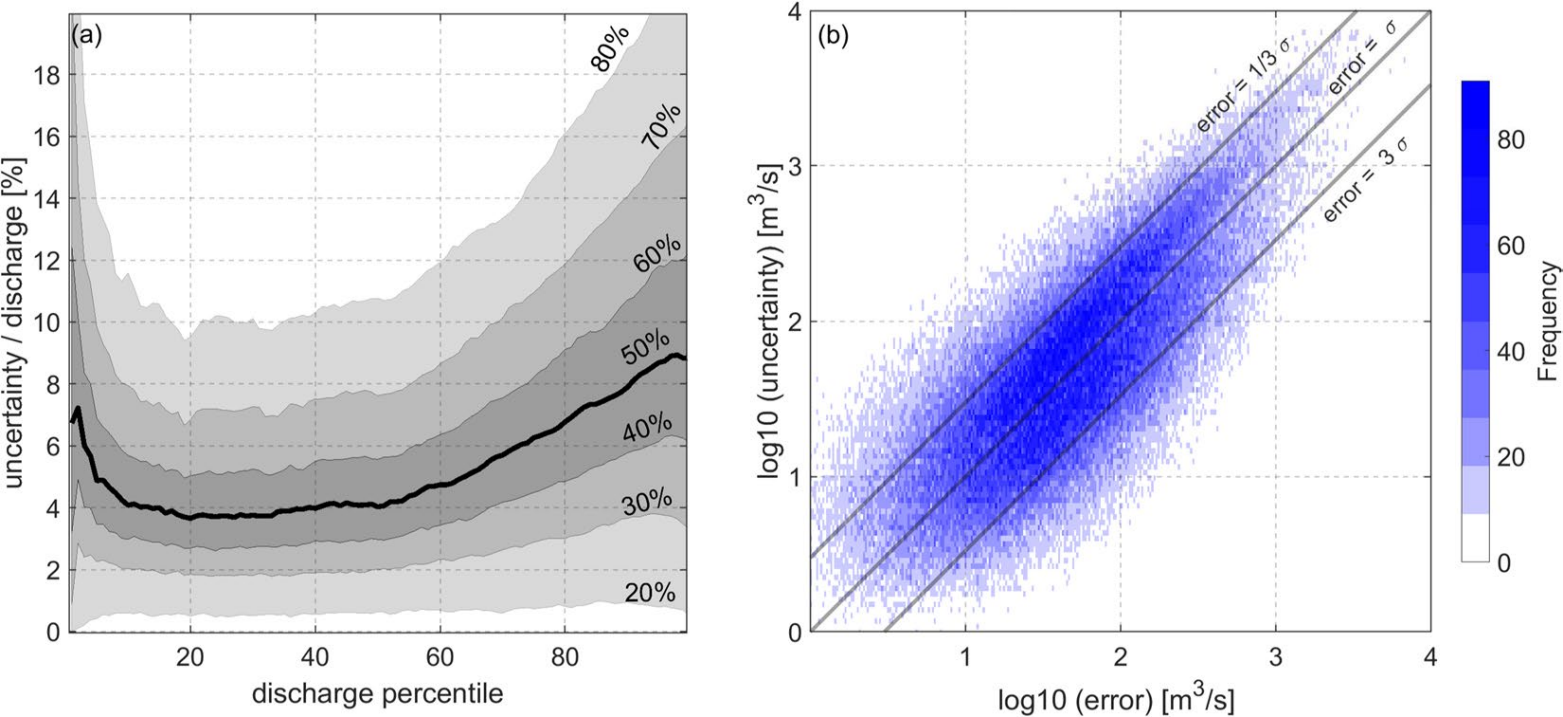
(**a**) Entire RSEG data set: Ratio of uncertainty to discharge across different discharge percentiles. The contour lines indicate the percentile of the ratio within each discharge percentile. The thick contour line represents the median of the ratio across each discharge percentile. (**b**) Validation set: histogram of estimated discharge uncertainty (*σ*) versus error (error = estimated discharge minus ground truth) for individual measurements across all stations in the validation set. To provide a better representation of data, the logarithm of both uncertainty and error are presented.

In addition, each time series is associated with a measure of uncertainty (shown as an error bar in Fig. 6), which includes both the remaining modeling error and the uncertainty associated with the input variables. Figure 7(a) shows the ratio of uncertainty to discharge plotted against corresponding discharge values at different discharge percentiles for the entire RSEG data set. So for each station within the RSEG data set, we determine the percentile of each discharge value through the empirical distribution of discharge time series specific to that station and calculate the ratio of uncertainty to discharge. Putting all these ratios for all stations (within RSEG data set) in one graph represented against the corresponding discharge percentile leads to Fig. 7(a), where the density of points is represented with a gray color. This visual depiction enables us to analyze and interpret the estimated uncertainty associated with different discharge percentiles. Our uncertainty estimate for lower discharge percentiles exhibits substantial variability between 0% to more than 20% of discharge values, with a median (the 50% percentile of the cases) value larger than 6%. Such a relatively large value (compared to discharge percentile between 20% and 50%) results primarily from increased uncertainty in the input data for lower flow situations where river channels tend to be narrower, where quantifying width or height from satellite imagery or altimeters becomes a difficult task. After the second discharge percentile, the ratio of uncertainty to discharge undergoes a reduction, consistently remaining within the range of 3–6% until reaching the 70th percentile. Subsequently, the ratio significantly increases and reaches 8% at the 90th percentile. This increment in the higher discharge percentiles can be attributed to the unavailability of sufficient river width measurements during high discharge events, mainly due to cloud contamination. In general, our uncertainty estimates are below the multiplicative uncertainty of 10% generally accepted in the scientific community. This analysis shows that the discharge uncertainty is not strictly multiplicative and also, the conventional 10% rule is so conservative.

## Data Records

The Remote Sensing-based Extension for the GRDC (RSEG) data set^20^ is freely available in the form of a NetCDF file on DaRUS, the Data Repository of the University of Stuttgart (10.18419/darus-3558). The data sets are available under the Creative Commons License: CC BY 4.0. Moreover, the discharge time series are also available on http://hydrosat.gis.uni-stuttgart.de.

The initial release of the RSEG data set comprises a total of 3377 discharge stations. Each station entry includes essential information such as the corresponding GRDC number and geographic coordinates. Furthermore, the climate classification, based on the Köppen-Geiger Climate Classification system, is provided for each station, offering valuable insights into the prevailing climatic conditions. Additionally, the data set incorporates the Pfafstetter code, a unique five-digit identifier derived from the HydroBasins data set^35^, associated with each station.

For a comprehensive analysis, the RSEG data set presents the discharge time series for each station, in units of m^3^/s, along with corresponding error estimates. The discharges are marked by three distinct flags, facilitating data interpretation: 0) In-situ discharge measurements, representing direct on-site measurements; 1) Remote sensing discharge estimates based on river height (water height time series obtained from DAHITI); 2) Remote sensing discharge estimates based on river height (water height time series obtained from HydroSat) and 3) Remote sensing discharge estimates based on river width (initial river mask time series obtained from JRC Global Surface Water, further enhanced by applying the Elmi & Tourian algorithm^29^). These flags enable researchers to discern the data’s origin and methodology. Moreover, each station has a quality flag representing the performance of the RS-based estimated discharge in the quality control procedure. If simultaneous gauge- and RS-based discharge data are available, the quality flag is marked as ‘good’ when the KGE value exceeds 0. If simultaneous data are unavailable, the quality flag will be marked as ‘good’ when the mean monthly correlation surpasses 0.5. The RSEG data set spans an extensive temporal range, with the earliest available in-situ measurement dating back to January 1806. The inclusion of remote-sensing data extends the record up to the end of 2022, offering an extensive data set for comprehensive hydrological investigations.

## Technical Validation

**Fig. 8:**
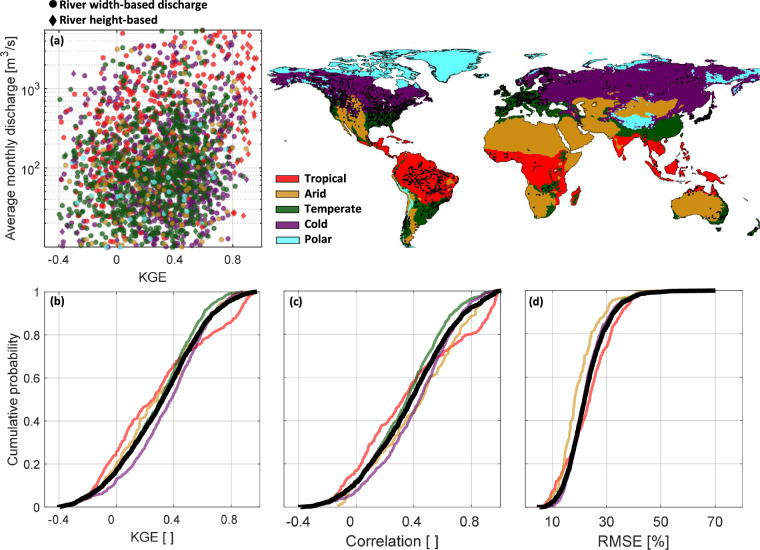
The map shows the location of the gauge stations used in the validation. (**a**) average monthly discharge vs. the Kling-Gupta Efficiency (KGE) between discharge records obtained from gauges and remote sensing data. (**b**–**d**) cumulative probability of three statistical metrics (KGE, correlation, RMSE) for the validation data set. The black lines in CDF plots are the validation for all gauges. Note that CDF plots are not represented for stations in the polar climate region due to the limited data set size.

For stations where estimated discharge and measured discharge are available simultaneously, it is possible to validate the results. However, such a simultaneous period should be long enough to yield reliable statistics. Therefore, we select those stations with at least 2 years of concurrent data, resulting in a set of 2168 stations with width-based discharge and a set of 219 stations with height-based discharge for validation. Figure 8 shows the KGE value of these stations together with their corresponding average monthly discharge for different climate classes. Moreover, Fig. 8 depicts Cumulative Distribution Function (CDF) plots, which effectively illustrate the distribution patterns of KGE, correlations, and relative RMSE across the validation sets. These plots serve to quantify the proportion of stations that exceed specific threshold values for each metric, providing insights into the performance characteristics. In general, about 80% of stations show a positive KGE (Fig. 8(b)), indicating that their estimated discharge is much better than the long-term mean. A comparative analysis between height-based and width-based approaches in Fig. 8 reveals that height-based discharge estimates, primarily associated with equatorial regions, outperform width-based ones.

Upon observing the khaki and coral results depicted in Fig. 8, it becomes apparent that the performance is comparatively inferior in arid and tropical climates compared with other climate classes. This can also be seen in Fig. 8(d), as about 35% of the stations show a relative RMSE larger than 30%. Whereas, the average performance of all stations in the validation set shows that only about 20% have relative RMSE values larger than 30%. The inferior performance in the equatorial region can primarily be attributed to the limitations of optical satellite imagery, specifically related to cloud contamination within the images.

Moreover, our analysis reveals that stations in cold climate regions perform marginally superior to stations in other climate classes (Fig. 8), in terms of KGE and correlation coefficients. This finding is particularly noteworthy due to the inherent difficulties associated with river width and height estimation in cold climate conditions, where rivers often experience freezing during the winter season and substantial fluctuations in river discharge occur during hot summers. The performance of the discharge estimation models depends significantly on the quality of input RS data. For larger river reaches, where we can obtain high-quality time series data for river width and height, the performance of the discharge estimation models is impressive, as indicated by a mean KGE value of 0.62 for stations with a mean monthly discharge exceeding 10000 m^3^/s. As shown in Table 1, a decrease in the mean monthly discharge is associated with a corresponding decrease in the performance of the discharge estimation models because obtaining high-quality time series data for river width and height in narrower river reaches is challenging.

To evaluate the validity of the uncertainty estimates and their applicability to the associated errors, we examine the relationship between uncertainty and error for all estimated discharges, as depicted in Fig. 7(b). To provide an overall assessment for all stations, we plot the logarithm of both values. The diagonal line in the histogram represents discharge estimates for which the provided uncertainty aligns with the outcome error. Moreover, two lines are shown below and above the diagonal line, signifying instances where the error is three times larger or smaller than our estimated uncertainty, respectively. The majority of the discharge estimates (83%) lie above the lower line, indicating that their error can be represented by estimated uncertainty with a confidence level of 99% (corresponding to 3*σ*). Nevertheless, in numerous instances (22%), our uncertainty estimates tend to become overly conservative. This is evident as certain measurements align with the upper line, where the error is three times smaller than the corresponding uncertainty.

The validation of our estimated discharge values and the associated uncertainty substantiates the adequacy of the RSEG data set in providing a fair estimate of discharge for 83% of worldwide river systems, as monitored by GRDC stations. The outcomes of our study will yield significant contributions to the understanding of Earth’s river networks as well as contributing to broader Earth system studies.

## Data Availability

The algorithm for developing the nonparametric quantile mapping function has been explained in detail in the original study^32^. The algorithm is developed in MATLAB and source code is provided together with the data set on DaRUS.
